# Stability of graph theoretical measures in structural brain networks in Alzheimer’s disease

**DOI:** 10.1038/s41598-018-29927-0

**Published:** 2018-08-02

**Authors:** Gustav Mårtensson, Joana B. Pereira, Patrizia Mecocci, Bruno Vellas, Magda Tsolaki, Iwona Kłoszewska, Hilkka Soininen, Simon Lovestone, Andrew Simmons, Giovanni Volpe, Eric Westman

**Affiliations:** 1 Division of Clinical Geriatrics, Department of Neurobiology, Care Sciences and Society, Karolinska Institutet, Stockholm, Sweden; 2 Institute of Gerontology and Geriatrics, University of Perugia, Perugia, Italy; 3 INSERM U 558, University of Toulouse, Toulouse, France; 4 3rd Department of Neurology, Memory and Dementia Unit, Aristotle University of Thessaloniki, Thessaloniki, Greece; 5 Medical University of Lodz, Lodz, Poland; 6 Institute of Clinical Medicine, Neurology, University of Eastern Finland, Kuopio, Finland; 7 Neurocenter, Neurology, Kuopio University Hospital, Kuopio, Finland; 8 Department of Psychiatry, Warneford Hospital, University of Oxford, Oxford, UK; 9 NIHR Biomedical Research Centre for Mental Health, London, UK; 10 NIHR Biomedical Research Unit for Dementia, London, UK; 11 Department of Neuroimaging, Centre for Neuroimaging Sciences, Institute of Psychiatry, Psychology and Neuroscience, King’s College London, London, UK; 12 Department of Physics, University of Gothenburg, Gothenburg, Sweden

## Abstract

Graph analysis has become a popular approach to study structural brain networks in neurodegenerative disorders such as Alzheimer’s disease (AD). However, reported results across similar studies are often not consistent. In this paper we investigated the stability of the graph analysis measures clustering, path length, global efficiency and transitivity in a cohort of AD (*N* = 293) and control subjects (*N* = 293). More specifically, we studied the effect that group size and composition, choice of neuroanatomical atlas, and choice of cortical measure (thickness or volume) have on binary and weighted network properties and relate them to the magnitude of the differences between groups of AD and control subjects. Our results showed that specific group composition heavily influenced the network properties, particularly for groups with less than 150 subjects. Weighted measures generally required fewer subjects to stabilize and all assessed measures showed robust significant differences, consistent across atlases and cortical measures. However, all these measures were driven by the average correlation strength, which implies a limitation of capturing more complex features in weighted networks. In binary graphs, significant differences were only found in the global efficiency and transitivity measures when using cortical thickness measures to define edges. The findings were consistent across the two atlases, but no differences were found when using cortical volumes. Our findings merits future investigations of weighted brain networks and suggest that cortical thickness measures should be preferred in future AD studies if using binary networks. Further, studying cortical networks in small cohorts should be complemented by analyzing smaller, subsampled groups to reduce the risk that findings are spurious.

## Introduction

Graph theory has become a popular tool in neuroimaging, providing methods to study complex brain networks^1^. These networks can be constructed from images of different modalities such as structural magnetic resonance imaging (MRI), functional MRI (fMRI), diffusion tensor imaging (DTI), positron emission tomography (PET) or electroencephalography (EEG)^2^. In the past few years, graph theory has been used to study the healthy and diseased human brain^3^. This technique allows automatic quantification of complex properties of networks in large cohorts and how these networks are altered in neurodegenerative disorders.

However, there are challenges associated with graph theoretical studies on the human connectome. Some of these challenges have been investigated in previous papers, which discuss inconsistent findings across studies^4–8^. There is the challenge of creating biologically meaningful networks by reducing voxel information into a sparse and discrete set of nodes^4^. Nodes and edge definitions vary across studies since no standard method exists yet in the graph theoretical community. The number of nodes in a network as well as choice of edge definitions have been shown to greatly influence graph properties^6,9^. Typically, nodes are defined from region of interests (ROI’s) specified by a neuroanatomical atlas and edge weights are often given by an over-simplified measure of association, such as Pearson correlation^4^. This measure has itself been shown to inherently give rise to small-world networks^5^. Thresholding is carried out to remove potential spurious connections in binary networks, but the specific threshold value might vary between groups and subjects. This gives the researcher a choice of either comparing networks of different densities (i.e. the ratio between the number of connected nodes and the number of possible connections), or comparing networks of the same density but at the risk of including spurious connections. This is a complicated issue since previous studies have shown that the density of a network can greatly influence network properties^9^ and that spurious connections can significantly impact graph metrics^10^. In weighted network analysis the issue of thresholding is avoided. However, the weight of an edge can be negative and most traditional measures require graph parameters to be between 0 and 1^1^. In such cases, researchers can make a choice between using absolute values or setting negative weights to zero. The former alternative only considers the magnitude of the negative weights, whereas the latter only (implicitly) makes use of the their sign. This choice is not trivial since the two approaches can lead to significantly different network properties. Normalizing with random networks has been proposed to overcome associated biases but finding an appropriate reference network is challenging since the reference network should be chosen based on both network measure and the measure of connectivity used to define the network edges^4^.

Alzheimer’s disease (AD) has been described as a disconnection syndrome characterized by network disruptions that seem to reflect the spread of pathological changes in the brain^11^. This makes graph theory an ideal tool for studying the progression of AD using different imaging modalities (see^12,13^ for review). While most papers assessing network disruption in AD have used EEG (e.g.^14,15^), fMRI (e.g.^16^), PET (e.g.^17^), or diffusion tensor imaging data (e.g.^18,19^), a few studies have investigated cortical network alteration in the continuum of AD using structural MRI data^20–27^. These studies have shown that both global and nodal network properties are altered in patients with AD compared to healthy controls. However, there is little agreement regarding the direction of these changes^6,7,13^. For instance, while some studies have shown an increase in the clustering coefficient in AD subjects^20,21^, others have shown a decrease of this network measure^23^ whereas a recent study found no significant global difference^28^. Further, the path length was significantly increased in AD subjects in some studies^20,21^, but decreased in another^29^. Phillips and colleagues showed that depending on edge definition *both* a significant increase and decrease in path length (as well as clustering) in AD subjects could be obtained^6^.

To our knowledge, no studies have assessed the influence of different atlases, anatomical measures or number of subjects on global network properties. The aim of this study is to investigate the impact these choices have on graph properties in relation to the differences between AD and control networks. Specifically, we tested whether similar results would be found when using two anatomical atlases of different resolutions to define nodes: the Desikan atlas^30^ (68 ROI’s) and the Destrieux atlas^31^ (148 ROI’s). In addition, we compared two types of networks–one based on cortical volumes and one on cortical thickness–and assessed how the number of subjects in each group would affect binary and weighted network findings. Understanding how these different choices affect graph measures derived from clinical data is crucial in order to correctly assess and compare graph theoretical findings. Investigating the impact group size and group composition have on network properties is of great importance in order to understand to what extent graph theoretical result acquired from a cohort can be generalized to a larger population. We hypothesized that there is a minimum number of subjects (referred to as MNS from here on) required to construct a group-based network and that this number is dependent on the heterogeneity of the group. When increasing the group size above the MNS value changes in graph properties should be negligible. We investigated the MNS value in AD groups and control groups and expected the MNS to be higher in the AD group, due to it being more heterogeneous, than in the healthy control group.

## Results

The graph properties studied were the *average strength* (the average cross-sectional correlation strength between brain regions), *characteristic path length* and *global efficiency* (measures of integration), together with *clustering* and *transitivity* (measures of segregation). Network properties of both weighted and binary graphs were computed. In the binary network analyses all measures were assessed over a set of network densities ranging from 5% to 35%, referring to the ratio between the number of connections in the network and the number of possible connections.

To assess the effect group size had on network properties, all measures were calculated for networks generated from groups of different sizes and group compositions. For each network measure, density (for binarized networks only), input measure, group size and composition, we used non-parametric permutation testing to determine if the difference between the subsampled AD and control groups was statistically significant with a two-tailed *p* < 0.05.

Characteristic path length was only considered for weighted networks since the binary networks were generally not fully connected at 35% network density. Further, since all pair of nodes in the weighted networks had non-zero edges the clustering coefficient become the same measure as transitivity. For more details, see Methods section.

### Effect of group size and composition

**Figure 1:**
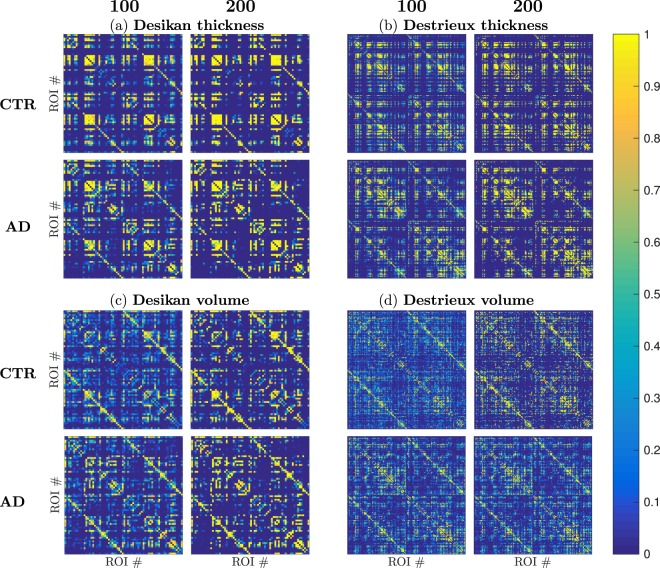
The average binary adjacency matrices of 1000 connectivity matrices binarized at a threshold corresponding to 15% network density when randomly subsampling 100 subjects (left) and 200 subjects (right) respectively. In each subfigure, the top row are the results from the control group (CTR) and the bottom row from Alzheimer’s disease group (AD). These connectivity matrices were constructed from different combinations of anatomical atlas and cortical measures (**a**–**d**). Each matrix element represents to the probability of two nodes being connected, ranging from 0–1 as shown by the colorbar.

An adjacency matrix is defined as a square matrix representing the nodes and edges of a graph. In a binary graph, a matrix element *A*_*ij*_ = 1 means that nodes *i* and *j* are connected. The average of 1000 binary adjacency matrices $\bar{A}$ for 100 and 200 randomly subsampled subjects are shown in Fig. 1. When ${\bar{A}}_{ij}$ = 0 nodes *i* and *j* were never connected. When ${\bar{A}}_{ij}$ = 1, *i* and *j* were connected for all group compositions. Regardless of atlas and input measures, increasing the group size made the edges in the average binary networks ${\bar{A}}_{ij}$ approach either 0 or 1. Since a value closer to 1 indicates a higher probability of two nodes being connected independently of group composition, this suggests that connections were less likely to be spurious due to a few particular subjects. As an example, the cortical thickness of left supramarginal and fusiform are only connected 62.7% of the times when creating a graph in the Desikan atlas from 100 AD subjects and 88.5% with 200 AD subjects in a binary network. The weighted graphs had only non-zero edges which led to that all nodes were connected.

We define the minimum number of subjects, MNS, as the number of subjects necessary for each graph property to converge to a final value. That is, further increasing the group size does not have a substantial effect on the given network property (see Methods for more detailed information). The MNS results and the discriminative abilities for each graph measure and construction methods are summarized in Table 1. AD group networks had lower MNS values, which implies that fewer AD subjects are needed to generate a network with stable properties than a network based on data from healthy controls. The MNS dependence of ROI and edge definitions varied depending on assessed graph measure and no combination of atlas and input measure decreased the MNS across all graph metrics. For both binary and weighted networks the MNS values were generally lower when using cortical thickness correlations as edges compared to volumes. However, the magnitude of the MNS values within the same network property showed no substantial agreement between binary and weighted graphs.

**Table 1:** Minimum number of subjects (MNS) needed for the average graph measure to be within ± 5% of the value for the full group network. The numbers in bold text denote that discrimination between controls (CTR) and AD was achieved with less than 293 subjects at the given density, atlas and input measure.

Graph measure, density	Desikan thickness (CTR/AD)	Destrieux thickness (CTR/AD)	Desikan volume (CTR/AD)	Destrieux volume (CTR/AD)
Average strength, weighted	**50**/**50**	**50**/**50**	**100**/**50**	**140**/**80**
Global efficiency, 15%	**135**/**50**	**150**/**50**	130/50	50/50
Global efficiency, 25%	**140**/**50**	90/50	50/50	50/50
Global efficiency, weighted	**50**/**50**	**90**/**50**	**130**/**90**	**170**/**140**
Transitivity, 15%	**120**/**50**	**125**/**60**	120/75	180/95
Transitivity, 25%	**105**/**50**	**135**/**55**	140/50	160/85
Transitivity, weighted	**50**/**50**	**50**/**50**	**60**/**50**	**130**/**50**
Clustering, 15%	50/50	60/50	200/75	200/160
Clustering, 25%	50/50	100/50	135/60	180/150
Clustering, weighted	**50**/**50**	**50**/**50**	**60**/**50**	**130**/**50**
Char. path length, weighted	**70**/**50**	**100**/**50**	**130**/**90**	**170**/**140**

**Figure 2:**
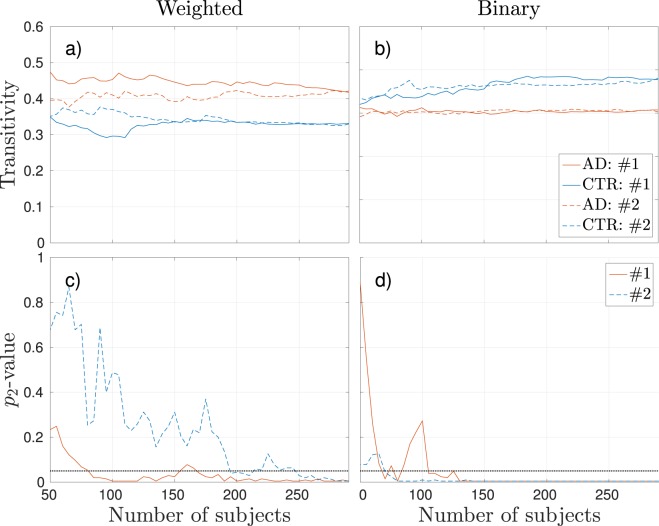
Transitivity computed from a weighted network (**a** and **c**) and a binary network at 25% density (**b** and **d**) when constantly adding five additional random subjects to each subgroup. Iteration #1 (solid lines) and Iteration #2 (dashed lines) represent two different iterations that started with 50 different random subjects in each group. The top plots show the calculated transitivity values for the two seeds when iteratively adding more subjects. The bottom plots show the respective corresponding two-tailed *p*-values computed with non-parametric permutations tests, where the dotted horizontal lines denote the threshold of significance of *p* = 0.05.

Figure 2 shows an example of how the transitivity measure could vary for different group compositions. Here we assessed what happens when we constantly add five more subjects to each subgroup and recompute the weighted (left) and binary (right) graph property. This means that all subjects used when forming the group of size 145 were also included in the group of 150 subjects. Depending on which specific subjects were used to create the group network, the results varied in significance when adding subjects to existing groups. In two specific iterations, #2 converged faster than iteration #1 for the binary case (Fig. 2b) and vice versa for the weighted (Fig. 2a). Hence, in iteration #1 for the binary network, the transitivity measurement would falsely indicate no differences between the AD and control groups of 100 subjects, while it would nonetheless indicate significant differences in iteration #2 with as few as 75 subjects.

**Figure 3:**
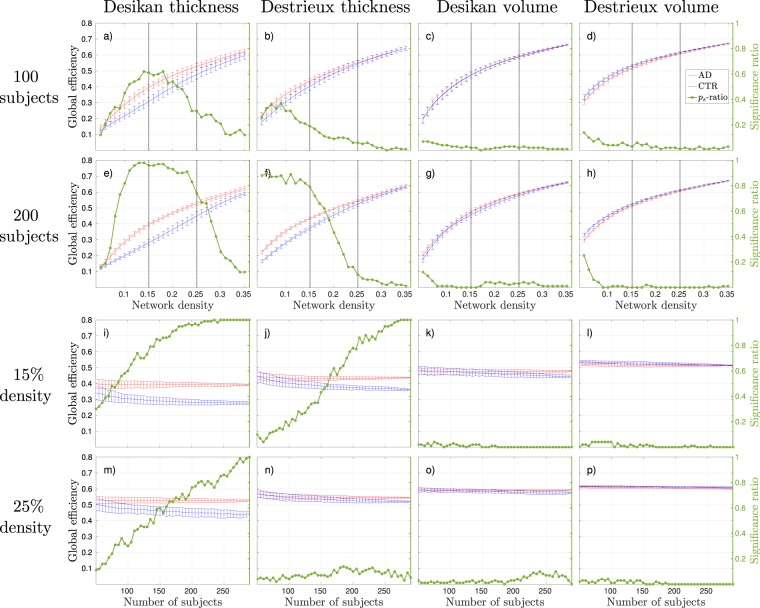
Global efficiency results, where blue lines correspond to control groups (CTR), red lines to Alzheimer’s disease (AD) patients, and the green line to the significance ratio (*p*_*s*_-ratio). The plots show the mean and standard deviations from 100 random group compositions and the ratio of significant 2-tailed *p*-values obtained from these random group compositions. (**a**–**d**) Results as a function of density with 100 subjects randomly subsampled. The horizontal dotted lines correspond to the network densities 15% and 25%. (**e**–**h**) Results as a function of density with 200 subjects randomly subsampled. (**i**–**l**) Results as a function of group size at 15% network density. (**m**–**p**) Results as a function of group size at 25% network density.

**Figure 4:**
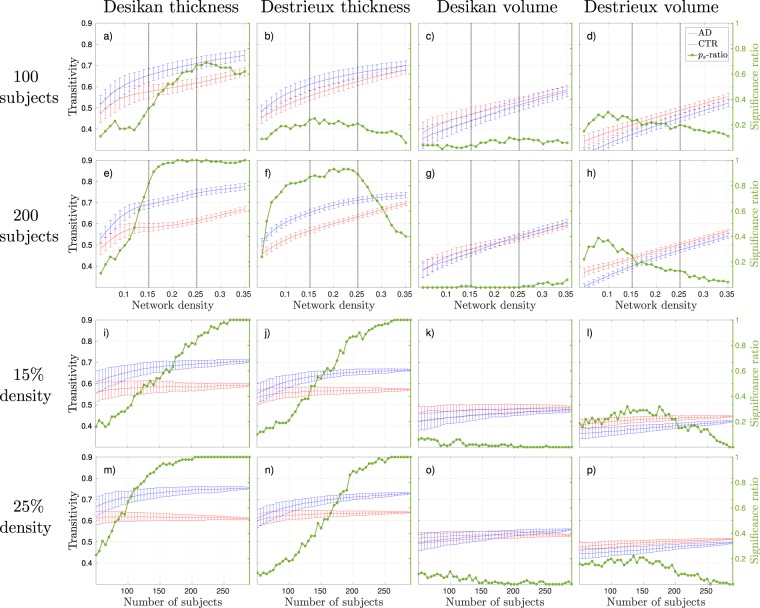
Transitivity results, where blue lines correspond to control groups (CTR), red lines to Alzheimer’s disease (AD) patients, and the green line to the significance ratio (*p*_*s*_-ratio). The plots show the mean and standard deviations from 100 random group compositions and the ratio of significant 2-tailed *p*-values obtained from these random group compositions. (**a**–**d**) Results as a function of density with 100 subjects randomly subsampled. The horizontal dotted lines correspond to the network densities 15% and 25%. (**e**–**h**) Results as a function of density with 200 subjects randomly subsampled. (**i**–**l**) Results as a function of group size at 15% network density. (**m**–**p**) Results as a function of group size at 25% network density.

**Figure 5:**
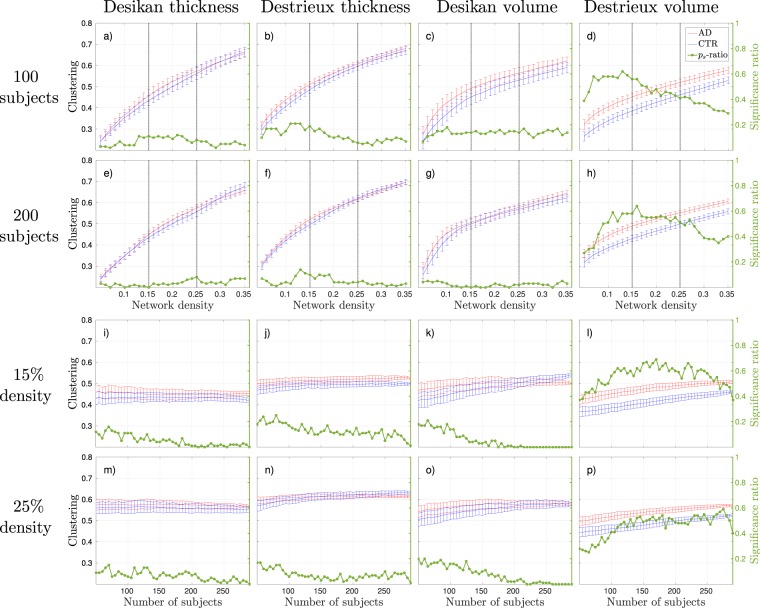
Clustering results, where blue lines correspond to control groups (CTR), red lines to Alzheimer’s disease (AD) patients, and the green line to the significance ratio (*p*_*s*_-ratio). The plots show the mean and standard deviations from 100 random group compositions and the ratio of significant 2-tailed *p*-values obtained from these random group compositions. (**a**–**d**) Results as a function of density with 100 subjects randomly subsampled. The horizontal dotted lines correspond to the network densities 15% and 25%. (**e**–**h**) Results as a function of density with 200 subjects randomly subsampled. (**i**–**l**) Results as a function of group size at 15% network density. (**m**–**p**) Results as a function of group size at 25% network density.

**Figure 6:**
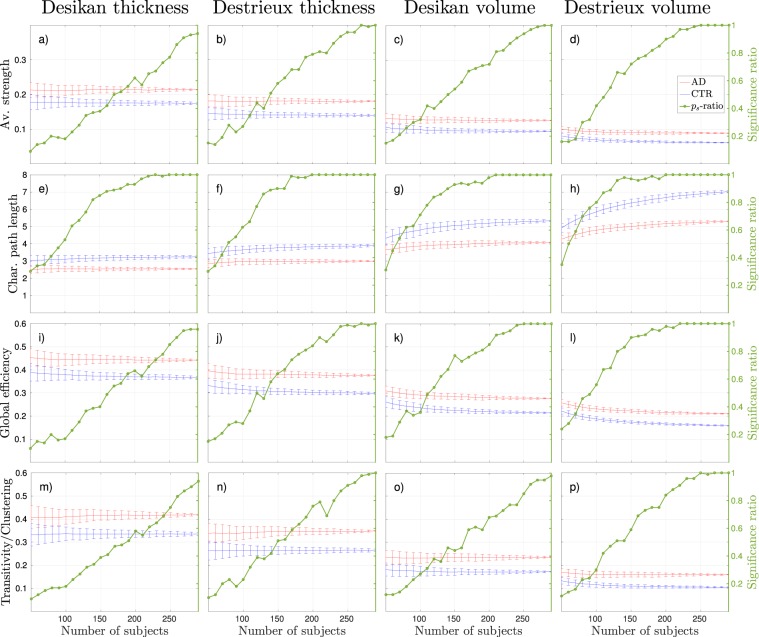
Results of weighted graph analysis, where blue lines correspond to control groups (CTR), red lines to Alzheimer’s disease (AD) patients, and the green line to the significance ratio (*p*_*s*_-ratio). The plots show the mean and standard deviations from 100 random group compositions and the ratio of significant 2-tailed *p*-values obtained from these random group compositions. The different columns represents different combinations of neuroanatomical atlas and cortical input measure. Each row shows the results of a different graph metric.

Focusing on the effects that group size have on binary networks in terms of discrimination, the general trend was that the significance ratio converged to either 0 or 1 when increasing the group size. The significance ratio refers to how often the 100 subsampled AD and control group compositions were found to be significantly different. The Figs 3, 4 and 5 show the mean and standard deviations of each binary graph metric from 100 different group compositions and how they varied as a function of density (a-h) and number of subjects used to construct the networks (i-p). The results from all weighted measures are shown in Fig. 6. The green lines (denoted as *p*_*s*_-ratio in the figures) illustrate the probability of acquiring a significant difference between the groups. We refer to this probability as the *significance ratio*. For global efficiency and transitivity around 175 subjects was required to obtain a statistical significant difference in 95% of the group compositions with the combination Desikan-thickness to form a binary network (Figs 3i and 4m). The corresponding value for Destrieux-thickness was 250, but the results in Fig. 3f suggest that the number of subjects required might be less at network densities <15%. For the weighted networks, the significance ratios converged to 1 with increasing group size for all measures. The characteristic path length was the measure that required the smallest group size in order to obtain reliable discriminative properties. As a Supplementary Analysis we present the corresponding weighted results that have been corrected for average strength by linear regression. These corrections removed the discriminative abilities for all weighted network measures–regardless of group size.

The MNS value for controls of the discriminative measures corresponded to a significance ratio between 0.5–0.9 for binary networks. The corresponding value for weighted networks were below 0.5 since the MNS values were generally lower for the weighted analyses, see Fig. 6 and Table 1.

### Effect of anatomical atlas and input measure

The general trend of the binary connectivity matrices seen in Fig. 1 was that the thickness measures (Fig. 1a,b) provided networks where more matrix elements tended to either 0 or 1 than the volumetric measures (Fig. 1c,d). We noted that at network densities of 15% and 25% a majority of the constructed networks had disconnected nodes, regardless of atlas and input measure. The average strength was stronger when using the thickness measures over volume measures and with the Desikan atlas compared to the Destrieux atlas which is shown in Fig. 6.

The effects of atlas and input measure were different for weighted and binary networks. In the case of binary graphs, the global efficiency was overall higher with the Destrieux atlas compared to the smaller Desikan atlas, whereas the transitivity was lower (see Figs 3 and 4). The same trend was observed for the thickness measures compared to the volume measures. The clustering coefficient results showed no distinct trend in terms of atlas size or input measure (see Fig. 5). For the weighted networks the trend was more consistent across measures. The combination Desikan-thickness yielded largest values, followed by Destrieux-thickness and lastly Desikan-volume and Destrieux-volume. This was true for average strength, global efficiency and transitivity (and thus clustering), but with the opposite direction for the characteristic path length.

In terms of discriminative abilities, the computed network properties were generally similar across the Desikan and Destrieux atlas. However, for binary networks the properties were highly dependent on input measure (see e.g. Fig. 4). The volumetric input yielded no significant differences between control and AD groups, regardless of network density and group size. In weighted networks we see in Fig. 6 that the discriminative abilities between AD and control groups (as well as the direction of the difference) were similar regardless of atlas and input measure. Surprisingly, the combination Destrieux-volumes required the fewest subjects to achieve significant difference regardless of group composition. However, if we factor out average strength from the other network measures using linear regression, the differences between the groups do not remain, which indicates that the average strength seems to be a dominating factor and driving the results of the other network measures. These adjusted results are presented as a Supplementary Analysis.

The global efficiency in binary graphs was found significantly increased in AD patients with networks generated from cortical thickness measures and regardless of atlas, see Fig. 3. The most significant network densities were in the range of [0.1, 0.25] when using the Desikan atlas (Fig. 3e), and [0.05, 0.15] with the Destrieux atlas (Fig. 3f). Transitivity provided the greatest discriminative abilities between control and AD groups for cortical thickness measures in binary networks (see Fig. 4). The most stable significant decreases in AD groups were found at densities _>_15% with the Desikan atlas (Fig. 4e) and at densities between [0.1, 0.25] with the Destrieux atlas (Fig. 4f). The number of subjects needed to acquire significant results in 95% of the group compositions with the Desikan atlas was 225 at 15% (Fig. 4i) and 150 at 25% network density (Fig. 4m). The corresponding numbers for the Destrieux atlas were 180 and 175 subjects. The clustering coefficient computed on binary networks did not show any promising results in terms of separating control groups from AD groups in this study, see Fig. 5.

## Discussion

Our study is the first to assess how different anatomical measures, node definition and group composition affect the stability and sensitivity of graph measures in structural brain networks derived from T_1_-weighted MRI images. We showed that all studied graph measures were sensitive to specific group composition within the same diagnostic group. These variations make it problematic for studies of smaller cohorts to draw conclusions without an inflated risk of acquiring a Type I or Type II error. Weighted network properties generally required fewer subjects to stabilize but the network properties were driven by the average correlation strength which limits the amount of additional information global network properties can provide. Further, our results showed similar differences between AD and control groups across atlases of different resolutions. In weighted networks the direction of these differences were maintained regardless of using cortical thickness or volume measures to construct the network. This was not the case for the binary networks, where only cortical thickness measures provided reliable significant differences. Our findings provide important clues that could potentially explain inconsistent results in previous binary network studies on AD and show promising stable features of weighted networks.

Contrary to our hypothesis, we found that the AD group required a smaller sample size in order to stabilize, i.e. showed a lower MNS value. The standard deviations for each group size were of similar order of magnitude between the control and the AD groups. This means that both groups were sensitive to group composition but that the AD group’s graph properties fluctuate around a fixed value, which it starts converging towards at a smaller group size. One explanation could be that the AD subjects showed stronger correlation patterns of atrophy which were not as prominent in the control subjects^32^. These patterns were registered by the Pearson correlation coefficient and became a dominant feature in the network and its properties. The average strength (i.e. the average correlations between cortical measures across subjects) was higher in the AD networks which supports that theory. The MNS values were lower than the number of subjects needed to reach a significance ratio of 0.95 in the discriminative measures. This suggests that the MNS value is too primitive to solely base a recommended minimum number of subjects on. We want the significance ratio to have converged at smaller group sizes than the MNS, if defined properly. The standard deviations of the different group compositions also need to be taken into account. This analysis suggests that the minimum number of subjects needed in each group to obtain robust results for binary vs weighted graphs were 150 and 130, respectively. These numbers will vary between cohort and research objective, and are likely dependent on the homogeneity within the group and of what magnitude the structural differences between the groups are. We recommend future studies comparing cortical networks between groups to complement their analysis with a subsampling scheme similar to the one described in this paper. That is, randomly select subsamples (without replacement) from each group and compute the graph properties of the networks. Repeat this process to assure that significant differences obtained when using full group sizes remain when removing a few subjects from each group.

On the effect of neuroanatomical atlas and number of ROI’s, Van Wijk *et al*. studied how path length and clustering varied in binary networks with increasing number of nodes *n* and fixed density^9^. The authors concluded that the clustering coefficient is relatively insensitive to network size whereas the non-normalized path length decreases with *n*. Our binary network results are in line with their findings when comparing the results from the Desikan (*n* = 68) and Destrieux atlas (*n* = 148) in terms of network size. No trend in the different atlas results was observed for the clustering coefficient but the global efficiency was increased in the larger Destrieux atlas. Seeing the global efficiency as the inverse counterpart to the path length, a decrease in path length with greater *n* infers an increase in global efficiency. Due to the similarity between clustering and transitivity equations it was expected that the transitivity results would behave in a similar way as the clustering coefficient across the atlases. However, there was a trend of decreasing transitivity with increasing *n*, albeit small compared to how the measure was affected by the choice of input measure. This could suggest that the decrease between atlases is due to the anatomical differences in how the ROI’s are defined rather than the number of nodes (the Desikan atlas is generated from gyri-based parcellation whereas the Destrieux atlas is constructed from a mixed sulco-gyral-based parcellation^31^). However, it is not possible to separate the effect of the different number of nodes from the effect of different parcellation schemes in our analyses. Studying the isolated effect of network size requires a systematic analysis on simulated data^9^. Investigating the isolated effect of parcellation scheme is more challenging but can potentially be done by comparing atlases defined through different anatomical landmarks but with the same number of ROI’s. Since modifying the atlas resolution would require also modifying the parcellation method it is difficult to investigate the combined effect of network size and different ROI definitions. A previous study has reported consistent relative differences between controls and epilepsy subjects in covariance networks across two atlases^33^. This, together with the results in this paper, may suggest that group network differences can persist across parcellation atlases, but not the actual graph measure values. However, more systematic studies on this topic are needed to draw any further conclusions, including what impact feature extraction using other software packages (such as SPM or FSL) have on graph properties.

The networks created using cortical thickness produced different results compared to cortical volumes. This is not surprising since the volume of a ROI, which can be seen as a combination of the thickness and the surface area, has been shown to be dominated by the latter^34^. Neither do cortical thickness and volume measure necessarily follow the same age-related decline^35^. Further, a recent study showed that when using cortical thickness, instead of volumes, different brain regions become important in terms of diagnostic separability in AD^36^.

In terms of discriminative properties between controls and AD networks, we observed statistically significant differences when using cortical thickness measures, whereas the volumetric measures only provided significant results for the weighted networks. One explanation for this can be that cortical thickness measures have been shown to better discriminate controls from AD subjects, and it is possible that they reflect the patterns of brain atrophy associated with AD better^37^. The average binary network results also showed that the volumetric measures were more sensitive to a specific group composition. From our results it is not possible to discard cortical volumes when studying binary structural networks alterations in AD. The low MNS value for the volumetric measures might still make it a good input measure for other neurodegenerative diseases. It is also possible that different graph construction methods and measures might show good discriminative abilities with cortical volumes.

The global efficiency metric has, to our knowledge, only been assessed once in gray-matter AD networks, where no significant difference was found between the control and AD group using volumetric input^28^. This is consistent with the results in our study. Most previous studies have opted for the path length using binary networks. Our results showed a stable and significant increase in global efficiency in AD which suggests a decrease in the path length: its inverse measure. However, previous studies on binary networks have typically showed an increase in path length^6,20,21^. These studies differ in several notable ways. Partial correlation was used to construct the networks in^20^, graphs were not fully connected in^6^, and volumetric input measures from a different atlas were used in^21^. Phillips *et al*.^6^ obtained both increased and decreased path length in AD for different graph construction methods. The fully connected weighted networks allowed for studying both characteristic path length and global efficiency. An interesting finding was that the weighted path length required the least number of subjects to obtain a reliable discrimination between control and AD groups. Contrary to the results derived from binary networks, cortical volumes defined by the Destrieux atlas provided the best discrimination for weighted measures. However, the MNS value was high and the increasing path length due to group size in Fig. 6h is not a desirable property in a stable and reliable network measure. A robust, significant decrease in transitivity in binary AD networks compared to controls was found in this study. The only two studies that have used transitivity to discriminate between controls and AD in structural gray-matter networks are on the ADNI data set^24^ or on the same combined cohort as in this study^23^. Not surprisingly–since also the same network construction methods were used–both studies showed a significant decrease in transitivity in the AD group compared to the control group. From the stability and discriminative properties of the transitivity measure shown in this study we recommend future studies on AD to use it. On the other hand, the weighted networks showed an increase in transitivity which means that the direction of changes is not consistent between binary and weighted networks. An increase of clustering in binary AD networks has previously been reported^20,21^, whereas a recent study obtained a decrease^23^, and another paper showed *both* an increase and a decrease depending on graph construction method^6^. In this study we did not observe any stable or significant differences in clustering between binary control and AD networks. Based on our findings, we advise future studies on binary gray matter networks to be particularly cautious when investigating the clustering coefficient of their networks.

Two of the most commonly reported graph properties in AD papers, path length and small-worldness, were not investigated in this project for binary graphs. The rationale behind this was that these measures are only meaningful in connected networks^1^, and the subsampled binary graphs in this study were generally disconnected at network densities ≤25%. A method to circumvent this issue is to only compute the measures on the fully connected subgraph within the network^38,39^. However, this would mean that when comparing two group networks it is likely that their respective (fully connected) subgraphs would be of different sizes and consist of different nodes. Since it has previously been shown that network size has a non-negligent effect on path length, it was deemed not meaningful to use these measures to compare topological properties for networks from different group compositions when these networks did not share the same nodes^9^. Therefore, the characteristic path length was only computed for weighted networks. An alternative to the characteristic path length measure is the global efficiency, related as the average inverse path length^1^. The limitations of the small-world measure in disconnected graphs have been discussed in e.g.^39,40^ where alternative measures based on efficiency instead of path length are proposed. We hope the brain network community adopts an alternative standard measure to the small-worldness measure that is not limited to fully-connected networks (see^41^ for a longer discussion). This would further increase the ability to compare results derived from different studies performing graph analysis in neuroimaging.

Comparing the stability and discriminative properties of binary and weighted graphs, we observed that both provide advantages and disadvantages in the analyses of AD networks. The weighted network measures required fewer subjects to stabilize (i.e. had a smaller MNS value) which would suggest that weighted networks are to be preferred when comparing groups with less than 150 subjects. However, the weighted network properties were driven by the average correlation strength, which implies that more complex information about the network is not gained. It further suggests that the choice of normalization schemes and covariate adjustments will have a large impact on the weighted network properties. Some measures, such as clustering and small-worldness, have several weighted definitions (e.g.^42–45^) with different properties that might solve this issue. We believe that the lower MNS value alone merits further investigations on weighted gray matter networks since they could possibly capture smaller structural changes (such as in preclinical AD^25,26^) better than binary networks because potentially important information from edge strengths is not lost^46^. On the other hand, binary networks have been more extensively investigated than weighted gray matter networks and are not dominated by the average strength^13^. This may imply that binary networks are more capable of capturing complex network patterns, but as new weighted brain network measures are redefined this could change in the future. Future studies using different cohorts and construction methods are needed to establish which approach (binary or weighted) should be preferred in brain network analyses as we can not know how our results would translate to different disease cohorts and construction methods.

The main limitation of this study was the finite sample size. To make the permutation tests between the subsampled groups unbiased, all subsampling was done without replacement. This causes a limitation in that the standard deviations are likely to decrease with increased group size by the mere fact that large groups are likely to have more overlap in subjects. For instance, when subsampling 290 out of 293 subjects two different group compositions will at least share 287 subjects which leads to a non-negligible decrease in the standard deviation compared to an infinite data set. The limited sample size probably also causes the MNS to be underestimated for the studied graph measures.

In this paper we have shown that group-based, cortical gray-matter networks are highly sensitive to which specific subjects are used to construct the connectivity matrix. These variations due to group compositions overpowered the difference between elderly controls and patients with Alzheimer’s disease, particularly at small group sizes. Our results suggest that more than 150 (130) subjects were needed to construct binary (weighted) group-based networks in this cohort of AD subjects and controls in order to reduce the risk of spurious findings. This recommendation is likely dependent on cohort and research question, but we believe that the systematic investigation presented in this paper adds to the picture of the behavior of gray matter networks and their properties. We advise future studies to rerun analyses on smaller, subsampled groups to ensure that significant results still are significant and thus more likely to be generalizable to a larger population. This study was carried out on a large cohort of controls and AD patients, but we believe that our findings are relevant to studies on other neurodegenerative disorders as well. Our study further showed, by comparing binary and weighted networks constructed from different parcellation atlases and cortical measures, that node and edge definitions have substantial effects on the graph properties. We believe that standardized methods in graph theoretical studies on brain networks would facilitate meaningful comparisons of findings across studies and greatly benefit the field.

## Methods

### Subjects

Data used in the preparation of this article were obtained from the Alzheimer’s Disease Neuroimaging Initiative (ADNI) database (adni.loni.usc.edu). The ADNI was launched in 2003 as a public-private partnership, led by Principal Investigator Michael W. Weiner, MD. The primary goal of ADNI has been to test whether serial magnetic resonance imaging (MRI), positron emission tomography (PET), other biological markers, and clinical and neuropsychological assessment can be combined to measure the progression of mild cognitive impairment (MCI) and early Alzheimer’s disease (AD).

AddNeuroMed is part of InnoMed (Innovative Medicine in Europe) and data was collected at six sites across Europe with the aim to develop novel biomarkers for AD and validate existing ones^47,48^.

In this project we combined data from ADNI and AddNeuroMed to increase the sample size. The inclusion and diagnostic criteria were similar in both studies. In short, to be eligible as a control a Mini Mental State Exam (MMSE) score > 24, a total Clinical Dementia Rating (CDR) of 0 and no signs of depression were required. The AD diagnoses were based on the criteria for probable AD of the National Institute for Neurological and Communicative Disorders and Stroke-Alzheimer’s Disease and Related Disorder Association (NINDS/ADRDA) together with CDR >0.5. For both groups, no history of drug abuse, organ failure, significant neurological or psychiatric illness (except AD) was required. No MRI data was used in order to make the diagnoses. Written informed consent was given by all participants (or by authorized representatives) in the ADNI and AddNeuroMed cohorts, in accordance with the Declaration of Helsinki. Both studies were approved by all participating centers’ respective institutional review board. The methods used in this study followed approved relevant guidelines and regulations. For more detailed information regarding inclusion and exclusion criteria for ADNI and AddNeuroMed, see e.g.^23,49^.

The demographics of the combined cohort are described in Table 2. It was important that the total number of control and AD subjects were equal in order to make the stability analyses comparable and unbiased. Since the number of control subjects included in the two cohorts was greater than the number of AD subjects, 43 out of the 336 controls were selected randomly and discarded from all further analyses to ensure equal group sizes of 293 subjects.

**Table 2:** Demographics of the studied cohort. The *p*-values provided are two-tailed and computed using permutation tests.

Variable	Controls	AD	*p*-value
ADNI - number of subjects:	197	172	—
AddNeuroMed - number of subjects:	96	121	—
Total number of subjects:	293	293	—
Age (years)	74.9 ± 5.7	75.5 ± 6.9	0.11
Gender (female ratio)	0.49	0.46	0.19
Education (years)	14.3 ± 4.4	12.0 ± 4.8	<0.001
MMSE score	29.1 ± 1.1	22.3 ± 3.7	<0.001
CDR score	0 ± 0	0.9 ± 0.4	<0.001

### MRI acquisition and image preprocessing

The AddNeuroMed study was designed to be compatible with the ADNI cohort^49^, and these two cohorts have successfully been combined in previous projects^23,50–52^. The acquisition and preprocessing of the MRI images followed the same procedure as in^50^. Briefly, all participants underwent a 1.5 T MRI scan acquired sagitally using a T_1_-weighted MPRAGE sequence with 9–13 ms repetition time (TR), 3.0–4.1 ms echo time (TE), 1000 ms inversion time (IT), 8° flip angle (FA) and voxel size of 1.1 × 1.1 × 1.2 mm^3^.

The T_1_ weighted images from the included subjects were preprocessed using the FreeSurfer 5.3.0 pipeline, freely available at http://surfer.nmr.mgh.harvard.edu/. In short, this involved motion correction and averaging^53^, removal of non-brain tissue^54^, intensity normalization^55^, Talairach transformation and segmentation of gray/white matter structures with automatic topology correction and optimal localization of tissue borders^56–60^. This was followed by deformation processes to register each subject to an individual spherical atlas based on cortical folding patterns^61^, parcellation of the cerebral cortex^62^ and calculating thickness and volumes measures^60^ from cortical regions specified by either the Desikan^30^ or the Destrieux atlas^31^. We extracted the cortical thickness and cortical volumes from 68 regions of the Desikan atlas and from 148 regions of the Destrieux atlas. All data was preprocessed through The Hive Database system (theHiveDB)^63^ and all output data was quality controlled.

### Network construction and graph analysis

The cortical thickness and volume measures extracted from FreeSurfer were used to define the nodes of the structural networks. Using the Desikan atlas, we built networks with 68 nodes, whereas for the Destrieux atlas we built networks with 148 nodes. The thickness and volume data from each region or node were corrected for the effects of age and gender using linear regression and the residual values were used to construct the connectivity matrices. For the cortical volumes, the intracranial volume was used as an additional covariate. The linear regressions were performed using data only from the control subjects. The reasoning behind generating a model based on controls only is that we wanted to detrend the effects of healthy aging and not the disease related changes we were interested in studying, as discussed in^50,64^. These residuals were used to calculate the edges between the nodes of the different atlases by computing the Pearson correlation between every pair of regions or nodes using the structural covariance method^65^. This resulted in 68 × 68 and 148 × 148 connectivity matrices for the Desikan and Destrieux atlas, respectively. For the binary networks, these matrices were binarized using a range of thresholds to ensure the networks had the same density (0.05–0.35, in steps of 0.01). At these network densities we noted that all correlations among the 35% strongest connections were positive (and greater than the absolute value of the smallest negative correlation) and therefore we did not consider the negative correlations. Following the procedure of a recent study, the absolute values of all correlations were used as weights in the weighted network analyses to avoid negative values^28^.

The global efficiency, clustering coefficient and transitivity were calculated from the binary networks across the different densities. For the weighted network analyses average strength, characteristic path length, global efficiency and transitivity were computed.

### Network measures

Since most network measures have both weighted and binary versions we denote measures only referring to weighted networks with a superscript *w* and analogously with a superscript *b* for binary networks. Measures that are defined the same for both binary and weighted networks have no superscript.

The characteristic path length is a measure of integration and is defined as

1 $${L}^{b}=\frac{1}{n}\sum _{i\in N}\frac{\sum _{j\in N,j\ne i}{d}_{ij}^{b}}{n-1},\,\,\,{L}^{w}=\frac{1}{n}\sum _{i\in N}\frac{\sum _{j\in N,j\ne i}{d}_{ij}^{w}}{n-1}$$

where *N* is the set of all nodes in the network, *n* is the total number of nodes, and ${d}_{ij}^{b}$ (${d}_{ij}^{w}$) is the shortest (weighted) distance between node *i* and *j* defined as

2 $${d}_{ij}^{b}=\sum _{{a}_{uv}\in {g}^{b}i\to j}{a}_{uv},\,\,\,{d}_{ij}^{w}=\sum _{{a}_{uv}\in {g}^{w}i\to j}f({w}_{uv})$$

with ${g}_{i\to j}^{b}$ (${g}_{i\to j}^{w}$) representing the shortest (weighted) path between the two nodes in the network computed using Dijkstra’s algorithm. The mapping *f*(*w*_*uv*_) is essentially the inverse (*w*_*uv*_)^−1^, but with *f*(0) = ∞. Since the *d*_*ij*_ is infinite for disconnected nodes it is not a meaningful global measure for disconnected graphs^1^. Instead, the global efficiency *E*^*b*^ (*E*^*w*^) can be used as a measure of integration where the inverse distance is used instead as

3 $${E}^{b}=\frac{1}{n}\sum _{i\in N}\frac{\sum _{j\in N,j\ne i}{({d}_{ij}^{b})}^{-1}}{n-1},\,\,\,{E}^{w}=\frac{1}{n}\sum _{i\in N}\frac{\sum _{j\in N,j\ne i}{({d}_{ij}^{w})}^{-1}}{n-1}$$

where an infinite nodal path length corresponds to nodal efficiency of zero^40^.

Clustering and transitivity are measures of segregation and quantify the presence of interconnected groups in a network. For binary networks these measures are based on the number of triangles around a node ${t}_{i}^{b}$ which corresponds to the number of closed loops where node *i* is connected to two nodes *j* and *k*, which are also connected to each other. The weighted analogue is based on the geometric mean of triangles around node *i* defined as ${t}_{i}^{w}=\frac{1}{2}{\sum }_{j,\in N}{({w}_{ij}{w}_{ih}{w}_{jh})}^{\mathrm{1/3}}$ which require ${w}_{ij} > 0$ for all *i*, *j* to avoid complex values of ${t}_{i}^{w}$. Clustering is described mathematically as

4 $${C}^{b}=\frac{1}{n}\sum _{i\in N}\frac{2{t}_{i}^{b}}{{k}_{i}({k}_{i}-1)},\,\,\,{C}^{w}=\frac{1}{n}\sum _{i\in N}\frac{2{t}_{i}^{w}}{{k}_{i}({k}_{i}-1)}$$

where *k*_*i*_ is the degree of node *i*, i.e. how many nodes it is connected to. The nodal degree *k*_*i*_ is defined in the same way for both weighted and binary networks as ${k}_{i}={\sum }_{j\in N}{a}_{ij}$, where *a*_*ij*_ = 1 if the connection strength is non-zero and *a*_*ij*_ = 0 otherwise. Transitivity is similar to the clustering measure and defined as

5 $${T}^{b}=\frac{\sum _{i\in N}2{t}_{i}^{b}}{\sum _{i\in N}{k}_{i}({k}_{i}-\mathrm{1)}},\,\,\,{T}^{w}=\frac{\sum _{i\in N}2{t}_{i}^{w}}{\sum _{i\in N}{k}_{i}({k}_{i}-1)}$$

The difference between these measures is that the clustering measure is normalized at nodal level, which makes it sensitive to nodes with lower degree, whereas the transitivity measure is normalized at a network level^1^. This is expected to make the clustering coefficient less robust in low density networks, which makes them interesting measures to compare in terms of stability. Note that in the special case in weighted networks where all edges are non-zero (leading to *k*_*i*_ = (*n* − 1) for all *i*), clustering is equal to the transitivity measures.

The network measures were computed using the Matlab based software BRAPH^24^, freely available at www.braph.org.

### Statistical analysis

For each density, we carried out non-parametric permutation tests to assess the difference between the control and AD subgroups with 50 subjects at a two-tailed *p*-value < 0.05. Once the graph measures were computed for all densities, five (ten for weighted networks) additional control and AD subjects were added to their respective subgroup and the network measures were computed again on the new networks. This procedure was repeated until 290 subjects had been added to each group. To examine the effects of group composition, the above procedure was repeated 100 times where 50 new random subjects from each group were subsampled as starting group. This means that both subsampling (to study the effect of group composition) and permutation tests (to assess the discriminative ability between the subsampled control and AD group) were used in the analyses.

The MNS value was computed for each graph measure, density and diagnostic group. It was defined as the smallest group size where the average network measure value was within ±5% of the full group size value. That is, increasing the group size would only cause the graph measure to fluctuate ±5% around a stable and representative group value. This definition assumes that the graph measure is stable and representative at the maximum group size of 293 subjects. It does not take into account how large the fluctuations due to group composition are, or how they affect the discriminative abilities between the control group and the AD group. To investigate these effects we used the two-tailed *p*-value from the permutation tests, computed for each density and group size over 100 randomized AD and control group compositions. This was performed in order to investigate the probability of obtaining significant differences (*p* < 0.05) for each group size, network density and graph measure, and thus the risk of making Type I and II errors.

### Data availability

Image data analyzed in the current study from Alzheimer’s Disease Neuroimaging Initiative (ADNI) database is publicly available, see adni.loni.usc.edu. The data from the The AddNeuroMed image is available from the corresponding author on reasonable request.

## Electronic supplementary material

Supplementary Analysis
