# Supplementary material for: Stability of graph theoretical measures in structural brain networks in Alzheimer’s disease
Source: Sci Rep. 2018 Aug 2;8:11592. doi: 10.1038/s41598-018-29927-0 (PMC6072788; doi:10.1038/s41598-018-29927-0)
Supplement: Supplementary file 1 — Supplementary Analysis [file 41598_2018_29927_MOESM1_ESM.pdf]

Supplementary Analysis: Stability of graph theoretical measures in structural brain networks in Alzheimer's disease

Gustav Mårtensson[1,*], Joana B. Pereira[1], Patrizia Mecocci[2], Bruno Vellas[3], Magda Tsolaki[4], Iwona Kłoszewska[5], Hilkka Soininen[6,7], Simon Lovestone[8], Andrew Simmons[9,10,11], Giovanni Volpe[12], and Eric Westman[1,11]

[1]Division of Clinical Geriatrics, Department of Neurobiology, Care Sciences and Society, Karolinska Institutet, Stockholm, Sweden.
[2]Institute of Gerontology and Geriatrics, University of Perugia, Perugia, Italy.
[3]INSERM U 558, University of Toulouse, Toulouse, France.
[4]3rd Department of Neurology, Memory and Dementia Unit, Aristotle University of Thessaloniki, Thessaloniki, Greece.
[5]Medical University of Lodz, Lodz, Poland.
[6]Institute of Clinical Medicine, Neurology, University of Eastern Finland.
[7]Neurocenter, Neurology, Kuopio University Hospital, Kuopio, Finland.
[8]Department of Psychiatry, Warneford Hospital, University of Oxford, Oxford, UK.
[9]NIHR Biomedical Research Centre for Mental Health, London, UK.
[10]NIHR Biomedical Research Unit for Dementia, London, UK.
[11]Department of Neuroimaging, Centre for Neuroimaging Sciences, Institute of Psychiatry, Psychology and Neuroscience, King's College London, London, UK.
[12]Department of Physics, University of Gothenburg, Gothenburg, Sweden.
[*]Corresponding author: gustav.martensson@ki.se

Weighted networks adjusted for average correlation strength

As supplementary information, we present the weighted network results that have been corrected for by the average network correlation strength (i.e. the average strength). This was done by first calculating regression slopes for each network measure, atlas and cortical measure, based on the measures presented in the main text. We regressed out the effect of average strength from all Alzheimer's disease (AD) and control (CTR) group network measures, together with the network measures from all the mixed-group networks generated in the permutation tests. These residual values were used to compute the significance ratios in the same way as described in the main article.

The corrected results and significance ratios are shown in Fig. 1. The minimum number of subjects (MNS) needed to obtain stable results are presented in Table 1. For the analogous unadjusted results of the weighted network analyses, see Fig. 6 and Table 1 in the main article. The adjusted results show practically no discrimination between control and AD groups for all computed graph metrics (Fig. 1). These results suggest that the properties of a weighted network are dominated by its average strength.

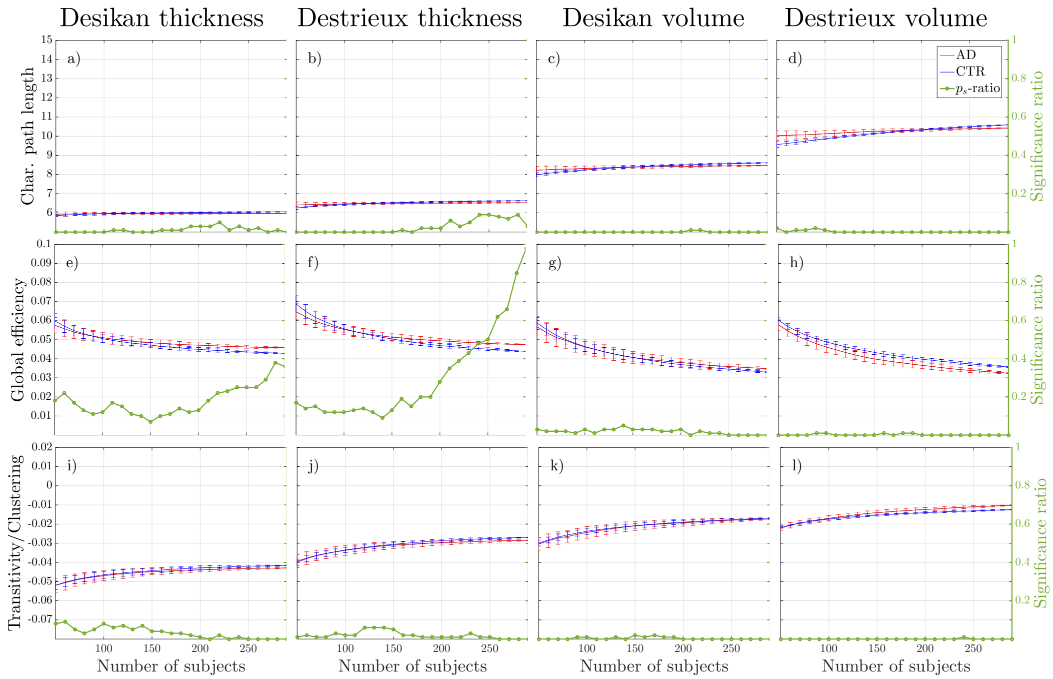

Figure 1. Results of weighted graph analysis where the effect of average strength has been adjusted for using linear regression. Blue lines correspond to control groups (CTR), red lines to Alzheimer's disease (AD) patients, and the green line to the significance ratio (p_s-ratio). The plots show the mean and standard deviations from 100 random group compositions (50 for the global efficiency measure on the Destrieux atlas) and the ratio of significant 2-tailed p-values obtained from these random group compositions. The different columns represents different combinations of neuroanatomical atlas and cortical input measure. Each row shows the results of a different graph metric.

Table 1. Minimum number of subjects (MNS) needed for the average weighted graph measure (adjusted for average strength) to be within $\pm$ 5% of the value for the full group network. The numbers in bold text denote that discrimination between controls (CTR) and AD was achieved with less than 293 subjects at the given density, atlas and input measure.

**Graph measure**	Desikan thickness (CTR/AD)	Destrieux thickness (CTR/AD)	Desikan volume (CTR/AD)	Destrieux volume (CTR/AD)
Global efficiency	170/140	180/160	290/190	290/290
Transitivity/clustering	290/290	290/290	290/290	290/290
Char. path length	50/50	50/50	70/50	110/50
